# Intramural Peri Arterial Drainage (IPAD) of peptides from the brain parenchyma and retina

**DOI:** 10.1002/alz70855_100903

**Published:** 2025-12-23

**Authors:** Roxana O. Carare

**Affiliations:** ^1^ University of Southampton, Southampton, United Kingdom

## Abstract

**Background:**

There are different pathways for the elimination of Cerebrospinal fluid (CSF) from the subarachnoid spaces of adult humans, including drainage across the cribriform plate into the nose. Interstitial fluid is eliminated from the brain along the basement membranes of capillaries and arteries as Intramural Periarterial Drainage (IPAD). Sporadic cerebral amyloid angiopathy (CAA) occurs as a result of a failure of Intramural Periarterial Drainage (IPAD) of amyloid‐beta (Abeta) along the walls of capillaries and arteries. The pattern of deposition of Abeta in the walls of cerebral and retinal arteries in Alzheimer's disease suggests that Abeta and solutes are eliminated from the retina along the basement membranes of retinal arteries, as IPAD.

**Method:**

Cadaveric retinae from patients with CAA were analysed for the presence of Abeta. Cadaveric cribriform plates were analysed for the presence of leptomeninges along the cribriform plate foramina

**Results:**

Abeta was identified in the basement membranes surrounding retinal artery smooth muscle cells. Epithelial membrane antigen, marker of leptomeninges, was identified as lining the cribriform plate foramina.

**Conclusion:**

This study demonstrates that in CAA, there is deposition of Abeta in the IPAD pathways, suggesting the failure of IPAD from the brain parenchyma is accompanied by a failure of retinal IPAD. The arachnoid as identified by positive staining for epithelial membrane antigen lines the foramina of the cribriform plates, providing thus a pathway for drainage from subarachnoid spaces into the nose.

